# Markedly poor physical functioning status of people experiencing homelessness admitted to an acute hospital setting

**DOI:** 10.1038/s41598-021-88590-0

**Published:** 2021-05-10

**Authors:** S. Kiernan, C. Ní Cheallaigh, N. Murphy, J. Dowds, J. Broderick

**Affiliations:** 1 Discipline of Physiotherapy, School of Medicine, Trinity College Dublin, University of Dublin, Dublin, Ireland; 2 Department of Physiotherapy, St. James’s Hospital, Dublin, Ireland; 3 Department of General Medicine and Infectious Diseases, St James’s Hospital, Dublin, Ireland; 4 School of Medicine, Trinity College Dublin, the University of Dublin, Dublin, Ireland

**Keywords:** Infectious diseases, Health services

## Abstract

Adults who are homeless experience poor health and frequently require hospital in-patient care but the physical functioning ability of this group is rarely considered. The objective of this study was to evaluate a broad range of physical functioning variables to enable better future planning of targeted health and accommodation services for this group. This cross-sectional, observational study was conducted in a large acute hospital in Dublin, Ireland. A comprehensive ward-based test battery evaluated physical functioning in 65 in-patients registered as homeless with an age range of 23–80 years. Less than 10% (n = 5) were > 70 years. 58/65 (83%) of participants had mobility limitations and 35/65 (54%) reported at least one fall in the previous six months. Only 25/66 (35%) were able to walk for 6 min and 20/65 (31%) were able to climb one flight of stairs. 45/63 (70%) of participants were pre-frail or frail. Muscular mass was normal in the majority of participants but grip strength was low. This study revealed hospital in-patients registered as homeless displayed particularly poor physical functioning levels and mobility regardless of age. Health and housing services should address the unmet physical functioning needs of this vulnerable group.

## Introduction

Homelessness is an extreme form of social exclusion^1^, which is globally relevant. Homelessness frequently occurs in individuals with high levels of childhood^2^ and adulthood trauma^3^, poverty^4^ and illiteracy^5^. A ‘person experiencing homelessness’ is someone who does not have stable housing who may live on the streets, in a shelter, or in other temporary, unstable or non-permanantlocations^6^. Figures indicate that 307,000 people are homeless in the UK^7^, 550,000 in the USA^8^ and 235,000 in Canada^9^ at any one time.

People who are homeless experience elevated rates of both chronic and acute disease, and frequently cope with the burden of multiple chronic medical conditions^10,11^ as well as mental illness and addiction^10^. Common chronic diseases such as chronic obstructive pulmonary disease, asthma, epilepsy, heart disease and stroke are experienced more frequently among people who are homeless compared to housed individuals^12^.

Unsurprisingly,given high levels of morbidity,people who are homeless utilize unscheduled acute hospital care disproportionally compared to housed individuals^13^. An emergency department visit rate of 60 times the rate of the general population was noted among the homeless population^14^ and high levels have been shown in other settings^15,16^. Rates of in-patient hospitalization of people who are homeless are also higher than those in stable housing^17–19^,with longer hospital stays and length of admissions^20,21^.

Homeless people who have been hospitalized may represent an especially vulnerable group with distinct care needs due to the complexity of their medical and social problems^22^, yet their physical profile has not been characterized.

Improved understanding of physical performance and functional limitation variables (such as strength, mobility, falls risk) of this group may help to identify ‘at risk’ individuals. These measures may give an indication of a person’s ability to perform everyday tasks making them good indicators of overall ability to live independently and are critical for targeting resource use and appropriate discharge planning^23^. This also provides an insight into early signs of disability, poor health and increased death risk^23,24^. Geriatric conditions such as functional impairments and falls are strongly associated with adverse health outcomes including acute care, institutionalization and death^25,26^ and manifest earlier in people experiencing homelessness^27,28^.

The objective of the study was to comprehensively profile adult in-patients registered as homeless using a broad battery of physical performance and functional limitation tests.

## Methods

This observational cross-sectional study took place in St. James’s Hospital which is the largest acute teaching hospital in Ireland. This hospital serves adults resident in the south inner city in Dublin,Ireland. The institutional ethical review board of Tallaght University Hospital/St. James’s Hospital approved this study. This study was performed in accordance with relevant guidelines/regulations and informed consent was obtained from all participants.

### Study procedure

During the hospital registration process conducted personally by hospital administration staff for all patients at the point of service entry,those identified as homeless (defined as lacking a stable place to live,and included individuals who were rough sleeping, sofa-surfing and those in short-term or long-term homeless accommodation) were flagged using the Microsoft Power B1 system. Following this, the clinical lead of the Inclusion Health Service,a consultant general physician (CNC), performed an initial eligibility screen of in-patients admitted to SJH, flagged as homeless, and accessed this group by checking the Power B1 dashboard, from November 2018 to May 2019, in St. James’s Hospital.

Definitive screening was undertaken by S.K. against the following inclusion and exclusion criteria. Inclusion criteria; (1) hospital in-patient, (2) homeless (3) > 18 years**.** Exclusion Criteria**;** (1) insufficient level of English to follow instructions required for study participation (unless translator present), (2) cognitive impairment,delirium,agitated state or other reasons to a degree that precluded assessment, (3) serious medical or orthopaedic reasons that would preclude ability to safely participate in some/all of test battery, (4 ) confirmed pregnancy.

Suitable patients were given a participant information leaflet and verbal information about the study. Study information was read aloud in plain English to accommodate participants with literacy issues. All participants gave written informed consent prior to participation in the study and following a process of rolling consent, they could terminate the assessment at any point. Each test was explained briefly and demonstrated to each participant by SK (a qualified physiotherapist familiar with the test battery). Further details of the test battery and methods are available in an earlier paper from this group^29^.

The following data was extracted from medical charts/ascertained from participants (1) baseline use of a mobility aid/wheelchair, (2) falls over the previous 6 months prior to admission.

### Test battery

1. To assess lower extremity physical function,the Short Physical Performance Battery (SPPB)^30^ was employed. This test comprises three tasks: a balance task,five timed chair stands,and a short timed walk. The scores range from 0 (worst performance) to 12 (best performance). The time (in seconds) taken to perform each component and the cumulative score were used to assess the results.
2. To screen for falls risk the Timed Up and Go (TUG) test was used^31^. The TUG measures the time it takes a person to stand up from an armchair,walk three meters and turn back to return to the chair^32^.
3. Functional capacity was estimated using the Six Minute Walk Test (6MWT). This test requires participants to walk up and down a thirty meter distance,over a six-minute period and the total distance covered is recorded^33^.
4. To estimate stair climbing ability as well as functional strength, balance and agility,the Stair Climb Test (SCT)^34^ was used. In this test, the time taken for participants to ascent and descent a stairs with 11 steps^35^ was recorded.
5. Frailty was measured using the Clinical Frailty Scale (CFS) which is scored on a scale from 1 (very fit) to 9 (terminally ill) and is based on clinical judgment^36^. Each point on the scale is correlated with a description of frailty along with a visual chart to aid the tester in classifying frailty.
6. To measure overall strength^37^, grip strength^38^ was measured using a Camry Digital Hand Dynamometer. This was performed in a sitting position while the hand was unsupported with the elbow at 90° flexion and the underarm and wrist in neutral positions. Three measurements were performed with each hand. An average of highest value for right and left sides was used in the analysis. Muscular mass was estimated by measuring mid-calf circumference using a tape measure.

### Statistical analysis

Descriptive analysis was undertaken to quantify physical functioning and performance of participants within this study. Statistical analysis was employed to (1) compare study variables between genders, (2) evaluate differences in study variables according to age divided into decades (ie 20 s,30 s,40 s etc.). Data was visually examined and plotted to evaluate for normal distribution and the presence of outliers. A 1-sample Kolmogoronov-Smirnof test was applied to assess for normality of distribution. If data were normally distributed independent t-tests were carried out to evaluate gender-based differences between variables and a Mann Whitney test was applied if data were non-normally distributed. To evaluate age-based differences for different decades of presentation, an ANOVA test was applied if data were normally distributed,a Kruskal Wallis test for non-normally distributed data. No adjustment was made for multiple testing. Data was assessed using IBM SPSS V24 and *p* < 0.05 was considered significant.

## Results

**Figure 1:**
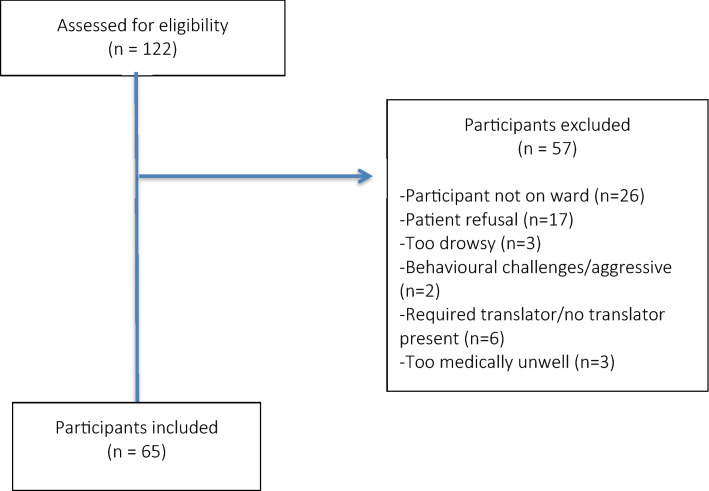
Flow diagram of participants through the trial.

The flow of participants through the study is shown in Fig. 1. Out of 122 patients assessed for eligibility, 57 were excluded for various reasons. The most prevalent reasons were that the patient was off the ward (despite re-visiting at least twice) (n = 23) or patient refusal despite revisiting patient where possible (n = 17).

Participant demographics are presented in Table 1. The majority of participants (n = 44, 67.7%) were male and the median (IQR) age was 45 (38, 56) years with a range of 23 to 80 years. Separated by biological sex, the median (IQR) of female participants was 41 years (33.5–46.5) and corresponding figure for male participants was 44 years (40.0–58.8). The majority of participants (n = 57, 87.7%) were born in Ireland. Most participants (n = 41, 64.0%) utilized hostel accommodation or were rough sleepers (n = 11, 17%). 34/65 participants (52.0%) were dependent on alcohol immediately prior to hospital admission and 23/65, (35.0%) were injecting or smoking heroin or crack cocaine immediately prior to admission. Many participants suffered from chronic medical conditions with hepatitis C (n = 27), liver disease (alcohol related) (n = 13) and epilepsy/seizure disorders (n = 11) and mental health conditions (n = 17) among the most common (Table 1).

**Table 1:** Demographic characteristics of participants.

Variable	N	%
**Sex**		
Male	44	66.7
Female	21	32.3
**Race/Ethnicity**		
White Irish	57	87.7
White non-Irish	5	7.7
African	2	3.1
Asian	1	1.5
**Medical conditions**		
Pancreatic disorders	6	9.3
Orthopedic disorders	6	9.3
Chronic obstructive lung disease	3	4.6
Liver disease (alcohol related)	13	20
Skin disease	5	7.7
Ulcers	5	7.7
HIV	7	10.8
Hepatitis	27	41.5
Seizures	11	16.9
Amputee	4	6.1
Depression	10	15.4
Bipolar disorder	4	6.1
Schizophrenia	3	4.6
Other mental disorder	2	3.0
**Current living arrangement**		
Hostel accommodation	41	64.1
Rough sleeping	11	16.9
With family/friends	5	7.7
Sheltered accommodation	3	4.6
Hotel	3	4.6
Rehabilitation facility	1	1.5
Unknown	1	1.5

Over thirty percent (n = 21) used some form of mobility aid and 8 participants used a wheelchair on a long term basis. The mobility aids included a stick (n = 6), crutches (n = 12) and a frame (n = 3).

### Compliance with physical performance battery

Participants completed some or all of the measures in the test battery as shown in Table 2. Compliance was lowest with the SCT (31.0%, n = 20) and the 6MWT (38.0%, n = 25). Two females terminated the 6MWT early, one at 30 m and one at 120 m. The entire test battery took approximately 30 min to complete and was completed in the same day where possible or within the same in-patient stay. Summary results of the physical test battery are presented in Table 2.

**Table 2:** Summary of physical performance measures.

Parameter	All participants	N	Male participants	n	Female participants	n	P value
Short Physical Performance Battery, median(IQR)	5(2–8)	42	5(2–8)	33	4(20–8.5)	9	0.902
Timed Up and Go, s, mean (SD)	22.39 (20.2)	36	18.4 (15.9)	27	33.9 (31.1)	9	0.058
Six Minute Walk Test, m, mean (SD)	289.9 (113.3)	27	293.2 (106.5)	18	235.7 (147.7)	9	0.257
Stair Climb Test, mean (SD)	22.7 (10.5)	20	22.1 (9.3)	15	24.5 (14.8)	5	0.677
Frailty, median (IQR)	4(3–5)	63	4(3–5)	42	4(4–6)	21	0.023*
Hand Dynamometry^1^, kg, mean (SD)	21.3 (10.5)	49	25.3 (9.1)	33	13.2 (8.5)	16	0.072*
Calf circumference, cm, mean (SD)	36.8 (5.1)	48	35.9 (4.4)	34	38.8 (6.2)	14	0.001*

^1^average of right and left sides, *p < 0.05.

### Lower extremity function

Out of 42 participants who completed the SPPB,half (n = 21, 50.0%) were unable to transfer from sitting to standing without using their hands for assistance. In addition, 26.0% (n = 11) participants scored zero in the balance subset as they could not hold a side-by-side stance for 10 s. Fifty percent (n = 21) were unable to transfer from sitting to standing without using their hands for assistance. There were no differences in SPPB scores between age and/or gender groups (*p* > 0.05).

### Falls risk

Of those who completed the TUG test,only 44.0% (n = 16) completed the test in > 13.5 s indicating that 56% (n = 20) were at a risk of falling. The mean (SD) age of those at risk of falling was 51 (15.2) years (range 27–80 years). One third (n = 12), took > 20 s to complete the test corresponding to low mobility levels. There were no differences between gender and/or age groups for completion time of the TUG (*p* > 0.05).

### Functional capacity

Of the 25 participants who completed the 6MWT, the mean distance covered was 289.9 m (111.3),with a range 30–505 m. There were no differences between age and/or gender groups for 6MWT distance (*p* > 0.05).

### Stair Climb Ability

In the 20 participants (31.0%) who completed the SCT the mean time taken was 22.7 s (10.5) (range 11.35–49.8 s). A 41-year-old female took the shortest time to complete the SCT (11.35 s),while a 29-year-old man took the longest (49.8 s). There were no differences in SCT scores between age and/or gender groups (*p* > 0.05).

### Frailty

Of the 63 participants who were scored on the CFS, only one participant obtained a score of 1 (very fit) and this participant was 80 years of age. In total,7 participants (11.1%) obtained a score of 2 indicating they were well, while 12 participants (19.0%) scored 3 meaning they were not regularly active but were managing well. The highest proportion of participants (n = 18, 28.6%) scored 4 indicating that while they vulnerable they did not depend on others for daily help.

A greater proportion of participants scored at the higher end of the scale with 39.7% (n = 25) classified as frail. Over thirty percent were pre-frail (n = 20, 30.8%). Overall, only 20 participants (31.7%) were classified as being robust or non-frail. The distribution of frailty scores was higher in females than males (*p* = 0.023) and there was no difference in frailty scores between age groups (*p* > 0.05).

### Grip strength

Out of the 16 females in this study the mean (SD) handgrip strength was 13.2 (8.5) kg, with the range 5.0 kg to 28.9 kg. The mean handgrip strength for males in this study was 25.3 (9.1) kg with a range of 7.2 kg to 41.4 kg. Males were significantly stronger than females *p* = 0.072. There were no differences in handgrip strength between age groups (*p* > 0.05).

### Calf circumstance

Calf circumference measurements were obtained for 48 participants (74.0%). The mean (SD) calf circumference of females in the study was 38.8 (6.2) cm (range 29.25 cm—51.5 cm) and for males it was 35.9(4.4) cm with a range of 23.5 to 45 cm. There were no differences in calf circumference measurements between male and female participants or between age groups (*p* > 0.05).

## Discussion

This appears to be the first study which employed a comprehensive battery to evaluate broad physical functioning variables of people experiencing homelessness who were in-patients in an acute hospital setting. Testing revealed a markedly high burden of physical and mobility limitations, irrespective of gender or age. The feasibility of the test battery was mixed as participation rates were low for a number of the standard tests extrapolated from the geriatric setting which were employed in this study (6MWT 38.0%, SCT 31.0%), with pain or not feeling well enough the commonest reasons for non-participation^29^.

SPPB testing revealed that the majority of participants (83.0%, n = 58), scored lower than 10, indicating one or more mobility limitations^39^. Half were unable to get out of a chair without using their hands for support,indicating a level of debility, and one quarter displayed extremely poor balance. This was backed up by the TUG test which revealed that over half (56.0%, n = 20) were at risk of falling and one third had low mobility^40^. It is unsurprising therefore; that almost one third used a mobility aid on a long-term basis and 8 participants used a wheelchair. Objective findings were supported by patient reporting that over half (54.0%) of this cohort experienced at least one fall in the previous 6 months. This highlights the need for falls prevention strategies. Our rate of falls is higher than values of 33.7% who reported a fall in the previous 6 months in an older cohort of community based sample homeless individuals (age > 50 years)^23^ and another study of formerly homeless housed individuals^41^.

A markedly low functional capacity was shown in study participants. The mean (SD) distance of the 6MWT covered in this study 289.9 m (111.3) which was considerably less than mean (SD) figures quoted for healthy individuals^42^ aged 55–75 years of 659 m (62). Only 30.0% of participants could attempt climbing one flight of stairs. The time per step was 1.03 s,which is about three times slower than figures quoted for a control population with a mean (SD) age of 58.3(12.4) years^43^.

Most participants (70.0%) were either pre-frail (vulnerable) or frail. Female participants displayed significantly higher frailty scores than male participants, despite a slightly lower median age in female participants (median age 41 female participants versus 44 years for male participants). This highlights potential additional vulnerability associated with females who are homeless which has been highlighted in previous work^11^.

Strength was also poor compared to normative values^44^. Reference average handgrip strength values^44^ for men aged 65–69 years of age, were 44.0 kg for males and 28.0 kg for women^44^. Our study in cohort with a much lower average age revealed a mean strength of 13.2 kg for female and 25.3 kg for male participants.

In the absence of advanced body imaging techniques to measure muscle mass or lean body mass, we simply measured mid-calf circumference, which can be prone to inaccuracies^45^. At odds with the rest of study variables, calf circumference measures were greater than normative cut off points of 34 cm in males and 33 cm in females^45^ in almost 90.0% of female participants and 70.0% of male participants. There are a number of possible reasons for this discrepancy. Firstly,the cut off values used^45^ were derived from an elderly population so are not directly comparable to the age distribution of our cohort. It is also possible that calf swelling may have been present due to intravenous drug injecting into the lower limb veins may have elevated lower limb circumference,limiting the ability to accurately measure this important construct of sarcopenia^46^. Notably,other criteria of sarcopenia such as low muscle strength and low physical performance^46^ were present in our study sample. We propose that measurement of mid upper arm circumference^47^ may be a useful measure to employ in future studies which evaluate a similar cohort.

Geriatric conditions such as functional impairment and falls were commonly observed in this study in agreement with previous studies^21,48,49^. Due to the high prevalence of functional and cognitive impairments,homeless adults can be considered “older” at age 50 years^23,48^. We extend this by reporting a far earlier onset of accelerated physical aging relative to the general population^50^. In agreement with other work^21^, we noted that chronological age was not a factor as expected age related decrements in physical functioning and performance did not apply. Our results showed that homeless individuals in their 20 s and 30 s experience geriatric syndromes also and poor physical status which was broadly consistent throughout the various age groups. Levels were comparable to elderly housed individuals^51,52^, highlighting the need to apply geriatric principles to manage the physical functioning needs of this population. As numbers of female participants were lower, it is possible that we lacked power to detect a difference in measures, with the exception of frailty,which should be explored in future studies.

The mean age of participants in this study at 47 years was similar to a previous study in this setting^15^ and likewise the majority of participants were male. Only 5 participants in this study were > 70 years, this under-representation of older in-patients may be partly due to earlier mortality^15^ among people who are homeless. Many participants suffered from co-existing medical conditions highlighting the complex medical needs of this population and the intensification of problems compared to an out-patient population of people experiencing homelessness^11^. Notably high was the number of participants in this study cohort who suffered from hepatitis (41.5%) which is approaching levels of 50.0% reported in a cohort of homeless men residing in Los Angeles^53^. It is likely that commonly reported medical conditions among people who are homeless such as headaches/migraines, hypertension and arthritis^54^ were under-reported among our study sample perhaps due to a lack of self-disclosure or diagnostic overshadowing. Respiratory problems^55^ were also likely under-reported especially as 80.0% of the cohort were current smokers which is comparable to rates over 70.0% quoted in community based homeless samples^50,56^. There was a high incidence of mental health conditions (29.0%), although as only established mental health diagnoses were considered it is likely that subclinical conditions such as anxiety were not represented.

Over half (52.0%) reported consuming excess alcohol and 20.0% had established alcohol related liver disease. Reports of alcohol abuse among community based homeless populations were lower than our study sample at 23.0%^55^, 21.0%^50^. One quarter of participants were reported to be active heroin users/intravenous drug which is likely to be underestimated^57^.

There were a number of limitations of this study. Firstly, this study collected data from one urban hospital setting so we are unsure if results are generalizable to wider homeless populations. Due to the cross-sectional nature of this study, we were unable to evaluate individual physical functioning trajectories over time. Participants were admitted to hospital, so for some mobility levels may have been temporarily lower than baseline levels. This needs to be considered in the interpretation of study results. We did not have accurate data on the length of time of homelessness, it is not known if length of homelessness worsens physical function and performance measures, although we suspect it does. Also deserving consideration is that a large percentage of in-patient admissions of people who are homeless are for psychiatric treatment^58^ which were not captured in this medical in-patient setting. The number of tests we included may have limited participation levels due to fatigue, although the full test battery took approximately 30 min to complete. A further limitation was that we excluded participants with likely severe impairment who were unable to attempt the test battery due to orthopaedic or medical reasons, so therefore this study may unwittingly be a snapshot of those with less severe symptoms, and may have underestimated true physical limitations. Finally, we did not explore the reason for poor mobility and physical status, which is likely to be a complex interplay of acute on top of chronic problems as well as multiple stressors and challenges associated with the homeless state.

Notwithstanding these limitations, strengths of this study were the reasonable sample size, and the broad clinically applicable test battery employed which was completed by a trained physiotherapist. By highlighting the markedly poor physical functioning of this cohort, this study makes an important contribution to the literature in a previously under-investigated area. A number of studies have reported medical health conditions of people^11,59^ who are experiencing homelessness but this study uniquely evaluated the physical manifestation of these medical conditions. Overall, a complex physical functioning burden was identified in this study. While this requires further evaluation, a challenge based on knowledge to date is to bridge the implementation gap^60^ and provide innovative, practicable, scalable solutions which are integrated into clinical practice and policy. At a minimum, people experiencing homelessness who are admitted to acute hospitals should be screened for possible physical rehabilitation interventions and referred appropriately. Some physical deterioration may be reversible which underlines the needs for early intervention services. Community referral pathways need to be established, ideally with outreach to homeless services and settings. Accommodation services for people who are homeless need to be accessible for those with low mobility levels. Future studies should determine suitable interventions to target physical health deficits and evaluate the physical functioning trajectory over time in this vulnerable group.

## Conclusion

People who are homeless have a much higher presentation and admission rate to the acute hospital setting than their housed counterparts and are likely to be a highly vulnerable group. Uniquely this study collectively evaluated this group using a broad range of robust physical functioning measures. Results indicated a notably high burden of geriatric conditions such as mobility and balance impairments, fragility, falls and low strength, irrespective of age, among people experiencing homelessness accessing in-patient hospital services.

Housing policy should respond to the physical health needs of people who are homeless and clinicians should screen for physical and mobility limitations. Referral to appropriate community and outreach services is needed as it is likely that ongoing support will be needed after discharge from hospital.
