# Publisher Correction: Markedly poor physical functioning status of people experiencing homelessness admitted to an acute hospital setting

**DOI:** 10.1038/s41598-021-96672-2

**Published:** 2021-08-23

**Authors:** S. Kiernan, C. Ní Cheallaigh, N. Murphy, J. Dowds, J. Broderick

**Affiliations:** 1 Discipline of Physiotherapy, School of Medicine, Trinity College Dublin, University of Dublin, Dublin, Ireland; 2 Department of Physiotherapy, St. James’s Hospital, Dublin, Ireland; 3 Department of General Medicine and Infectious Diseases, St James’s Hospital, Dublin, Ireland; 4 School of Medicine, Trinity College Dublin, The University of Dublin, Dublin, Ireland

Correction to: *Scientific Reports* 10.1038/s41598-021-88590-0, published online 10 May 2021

The original version of this Article contained an error in the Introduction, where

“Figures indicate that 307,000 people are homeless in the UK^7^, {, #2306}{, 1986 #2260}{, #2306}{, #2306}, 550,000 in the USA^8^ and 235,000 in Canada^9^ at any one time.”

now reads:

“Figures indicate that 307,000 people are homeless in the UK^7^, 550,000 in the USA^8^ and 235,000 in Canada^9^ at any one time.”

The original Article has been corrected.

